# Drug-Resistant Tuberculosis, Lebanon, 2016 – 2017

**DOI:** 10.3201/eid2503.181375

**Published:** 2019-03

**Authors:** Salam El Achkar, Christine Demanche, Marwan Osman, Rayane Rafei, Mohamad Bachar Ismail, Hiam Yaacoub, Claire Pinçon, Stéphanie Duthoy, Frédérique De Matos, Cyril Gaudin, Alberto Trovato, Daniela M. Cirillo, Monzer Hamze, Philip Supply

**Affiliations:** Université de Lille, CNRS, INSERM, Centre Hospitalier Universitaire, Institut Pasteur de Lille, U1019–UMR 8204–Centre d’Infection et d’Immunité de Lille, Lille, France (S. El Achkar, C. Demanche, C. Pinçon, P. Supply);; Université Libanaise, Tripoli, Lebanon (S. El Achkar, M. Osman, R. Rafei, M.B. Ismail, M. Hamze);; Genoscreen, Lille (S. Duthoy, F. De Matos, C. Gaudin);; IRCCS San Raffaele Scientific Institute, Milan, Italy (A. Trovato, D.M. Cirillo);; Ministry of Public Health, Beirut, Lebanon (H. Yaacoub)

**Keywords:** Tuberculosis, drug resistance, survey, DNA sequencing, whole-genome sequencing, refugees, asylum seekers, migrant workers, TB, MDR, XDR, tuberculosis and other mycobacteria, bacteria, Lebanon, antimicrobial resistance

## Abstract

In a 12-month nationwide study on the prevalence of drug-resistant tuberculosis (TB) in Lebanon, we identified 3 multidrug-resistant cases and 3 extensively drug-resistant TB cases in refugees, migrants, and 1 Lebanon resident. Enhanced diagnostics, particularly in major destinations for refugees, asylum seekers, and migrant workers, can inform treatment decisions and may help prevent the spread of drug-resistant TB.

Populations in crisis-affected areas are particularly vulnerable to tuberculosis (TB) linked to malnutrition, overcrowding, and discontinuity in health services (*1**,**2*). Difficulties accessing diagnosis and starting or completing appropriate treatment can promote the emergence and spread of multidrug-resistant (MDR) TB (resistant to at least rifampin and isoniazid) and extensively drug-resistant (XDR) TB (additionally resistant to >1 second-line injectable drug and 1 fluoroquinolone) in the countries of origin or in countries of transit or refuge after migration (*3*).

Lebanon hosts the largest per capita refugee population in the world. In addition to 450,000 refugees from Palestine, ≈1.5 million refugees from Syria are scattered in hundreds of informal sites across the nation (*2**,**4*). Moreover, the country hosts >250,000 migrant domestic workers, mostly originating from regions with high TB incidence rates, such as Ethiopia, Bangladesh, the Philippines, and Sri Lanka (*5*).

The last national survey on the prevalence of drug-resistant TB in Lebanon was performed 15 years ago (*6*), well before the beginning of the Syria crisis in 2011. Even most recent reported MDR TB rates largely relied on estimates rather than on systematic laboratory confirmation (*6*). Second-line drug susceptibility testing (DST) and individualized XDR TB treatments were not available. We report results from a June 2016–May 2017 nationwide study combining extensive phenotypic and molecular testing. This national survey was approved by the ethics committee of the Azm Center for Research in Biotechnology and Its Applications, Lebanese University (document no. CE-EDST-3-2016), authorized by the Lebanese Ministry of Public Health. Informed consent was obtained from the study patients. 

## The Study

The study included 720 cases of suspected TB, corresponding to all suspected cases reported from June 1, 2016, through May 31, 2017, to the TB centers from the 9 governorates that make up Lebanon’s national TB program. After testing of all corresponding microscopy-positive and microscopy-negative samples, 284 were considered confirmed TB cases on the basis of solid (Lowenstein-Jensen [LJ]) or liquid (BBL MGIT Mycobacteria Growth Indicator, BD Diagnostics, http://www.bd.com) culture results or molecular testing results (Xpert MTB/RIF, Cepheid, http://www.cepheid.com). For samples contaminated with blood, Anyplex MTB/NTM Real-time Detection (Seegene, http://www.seegene.com) (Appendix 1) was used. Thirty-four cases could not be subjected to DST because of culture negativity (n = 28), contamination (n = 3), insufficient sample amount for culture (n = 2), or reagent contingencies (n = 1). 

Of the 250 remaining patients, 51% (128/250) were men; the mean age was 34 years (Table 1; Appendix 2). Patients were from Syria (74/250, 29.6%), Lebanon (70/250, 28%), Ethiopia (57/250, 22.8%), Bangladesh (13/250, 5.2%), Palestine (7/250, 2.8%), or other nations (29/250, 11.6%).

**Table 1:** Details of 250 TB cases with available phenotypic drug susceptibility profiles, Lebanon, 2016–2017*

Characteristic	No. (%) patients			
	Total, n = 250	New cases, n = 228	Previously treated or relapsed, n = 18	Missing data, n = 4
Sex				
M	128 (51.2)	112 (49.1)	14 (77.8)	2 (50)
F	122 (48.8)	116 (50.9)	4 (22.2)	2 (50)
Country of origin				
Lebanon	70 (28)	64 (28.1)	4 (22.2)	2 (50)
Syria	74 (29.6)	65 (28.5)	9 (50)	0
Ethiopia	57 (22.8)	54 (23.7)	1 (5.6)	2 (50)
Bangladesh	13 (5.2)	13 (5.7)	0	0
Palestine	7 (2.8)	7 (3.1)	0	0
Other	29 (11.6)	25 (11)	4 (22.2)	0
Age, y	34 ± 14	34 ± 14	38 ± 13	23 ± 5
Drug resistance				
RIF	7 (2.8)	3 (1.3)	4 (22.2)	0
Mono	1 (0.4)	1 (0.4)	0	
MDR	3 (1.2)	1† (0.4)	2‡ (11.1)	
XDR	3§ (1.2)	1§ (0.4)	2§ (11.1)	
INH	16 (6.4)	15 (6.6)	1 (5.6)	0
Mono	9 (3.6)	8 (3.5)	1 (5.6)	
INH + SM	7 (2.8)	7 (3.1)	0	
EMB only	1 (0.4)	1 (0.4)	0	0
SM only	23 (9.2)	21 (9.2)	2 (11.1)	0
Susceptible to all first-line drugs	203 (81.2)	188 (82.4)	11 (61.1)	4 (100)

*Age is expressed as mean ±SD; categorical variables are presented as absolute numbers and percentages. EMB, ethambutol; INH, isoniazid; MDR, multidrug resistant; mono, monoresistant; RIF, rifampin; SM, streptomycin; XDR, extensively drug resistant.  †Resistant to RIF and INH. ‡Resistant to RIF, INH, EMB, and SM. §Resistant to RIF, INH, EMB, SM, amikacin and kanamycin, and levofloxacin (representing all tested drugs for MDR and XDR isolates).

Rifampin resistance was detected among 7/250 (2.8%) patients, concordantly with Xpert testing results for all cases (Table 1). We used multivariate logistic regression to test TB history as an independent predictor of rifampin resistance, after adjusting for age, sex, and nationality (Appendix 1). Log-linearity was checked for age. A 2-tailed type I error rate was set at 5%. TB history information was available for 246 (98.4%) patients. The proportion of rifampin resistance was 22.2% (4/18) among previously treated patients and patients with relapse and 1.3% (3/228) among patients with new TB cases (adjusted OR 21.4, 95% CI 4.4–105.2; p<0.01). One case in a patient without previous TB history was confirmed by liquid culturing DST as monoresistant to rifampin; 3 other cases, including 1 in a patient without previous TB history, were MDR TB, 2 of which showed resistance to all 4 first-line drugs tested (i.e., ethambutol and streptomycin in addition to rifampin and isoniazid). Moreover, 3 XDR TB cases were detected, including 1 in a patient without previous TB history, showing phenotypic resistance to amikacin, kanamycin, and levofloxacin in addition to all 4 first-line drugs tested. Among all 250 cases, 203 (81.2%) were susceptible to all 4 first-line drugs, 9 (3.6%) were resistant to isoniazid only, 1 (0.4%) to ethambutol only, 23 (9.2%) to streptomycin only, and 7 (2.8%) to isoniazid and streptomycin (Table 1).

To assess their extensive drug-resistance profiles, we subjected isolates from the 3 patients with XDR TB to targeted sequencing by use of a new assay, Deeplex-MycTB (GenoScreen, https://www.genoscreen.fr), which covers 18 drug resistance–associated gene targets (*7*) (Figure; Appendix 1). Two of these cases were confirmed by whole-genome sequencing. In 1 case (patient identification no. 74), no mutation was found to explain phenotypic resistance to amikacin and kanamycin. For the other drugs for this isolate, and for the 2 isolates analyzed by both tests, we detected drug resistance–associated mutations (*8**–**10*) in *rpoB*, *katG* or *inhA*, *gyrA*, *rrs* or *tlyA*, and *embB*, confirming the resistance phenotypes (Table 2). Moreover, we detected different drug resistance–associated deletions in *ethA* in all 3 XDR TB isolates and drug resistance–associated mutations in *pncA* in 2 XDR TB isolates. These mutations predict additional resistance to ethionamide and pyrazinamide, which are not phenotypically tested in Lebanon or in many other countries.

**Figure:**
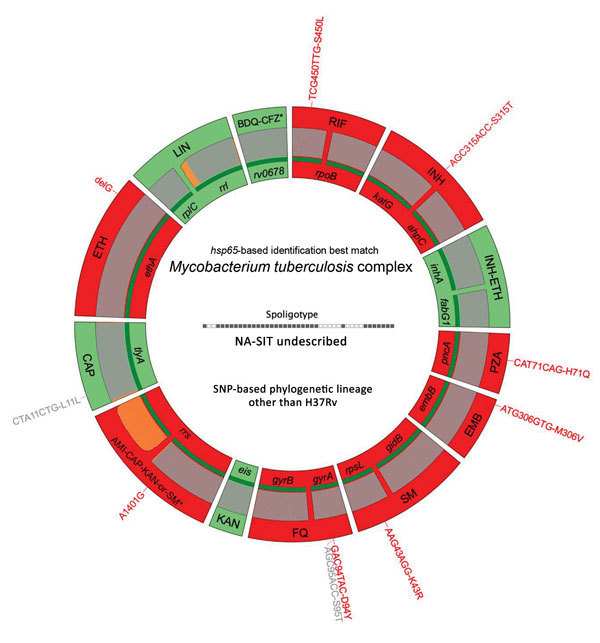
Deeplex-MycTB (GenoScreen, https://www.genoscreen.fr) results identifying an extensively drug-resistant genotypic profile in an isolate from a tuberculosis (TB) patient in Lebanon. Results correspond to TB patient no. 185 in Table 2. Target gene regions are grouped within sectors in a circular map according to the drug resistance with which they are associated. Red indicates target regions in which drug resistance-associated mutations are detected (red text around the map), whereas green indicates regions where no mutation or only mutations not associated with resistance (gray text around the map) are detected. Dark green lines above gene names represent the reference sequences with coverage breadth above 95%. Limits of detection (LOD) of potential heteroresistance (reflected by subpopulations of reads bearing a mutation), depending on the coverage depths over individual sequence positions, are indicated by gray (LOD 3%) and orange (variable LOD >3%–80%) above the reference sequences. Information on mycobacterial species identification, based on *hsp65* sequence best match, and genotype of *Mycobacterium tuberculosis* complex strain, based on spoligotype and lineage-defining phylogenetic SNP, are shown in the center of the circle. AMI, amikacin; BDQ, bedaquiline; CAP, capreomycin; CFZ, clofazimine; EMB, ethambutol; ETH, ethionamide; FQ, fluoroquinolones; KAN, kanamycin; LIN, linezolid; INH, isoniazid; PZA, pyrazinamide; RIF, rifampin; SM, streptomycin; SNP, single-nucleotide polymorphism.

**Table 2:** Genotypic and phenotypic drug susceptibility profiles of drug-resistant TB cases, Lebanon*

Category			Drug resistance classification						
			MDR				XDR		
Patient ID			14	48	125†		74	168	185
TB drug									
RIF	Gene	*rpoB*	S450L	S450L	S450L		S450L	**S450L**	**S450L**
	Phenotypic								
INH	Genes	*katG*	S315T	F129S‡	S315T		S315T		**S315T**
		*inhA*						**C-15T**	
	Phenotypic								
PZA	Gene	*pncA*		Y103Stop	InserA192		A46P		**H71Q**
	Phenotypic		ND	ND	ND		ND	ND	ND
EMB	Gene	*embB*	Q497R	M306V	M306V		Q497R	**Q497R**	**M306V**
	Phenotypic								
SM	Genes	*rpsL*	K43R		K88R		K43R		**K43R**
		*rrs*						**A908C**	
	Phenotypic								
AMI/KAN	Gene	*rrs*						**A1401G**	**A1401G**
	Phenotypic								
FQ	Gene	*gyrA*		S91P§			D94A	**S91P**	**D94Y**
	Phenotypic								
CAP	Genes	*tlyA*					InserC313		
		*rrs*						**A1401G**	**A1401G**
	Phenotypic		ND	ND	ND		ND	ND	ND
ETH	Genes	*ethA*	Deleted¶		141 nt del#		Deleted¶	**DelG632**	**DelG1338**
		*inhA*						**C-15T**	
	Phenotypic		ND	ND	ND		ND	ND	ND
MIRU-VNTR type**			100-32	19431-157	21404-32		10156-32	21416-15	?-15
*M. tuberculosis* complex lineage††			2 (Beijing)	4 (Euro-American)	3 (Delhi-CAS)		2 (Beijing)	4 (H37Rv-like)	4 (Haarlem)

*Only genes with detected resistance-associated mutations are shown. No mutation was detected in targets associated with linezolid or bedaquiline and clofazimine resistance. Mutations are shown as amino acid changes with the corresponding codon position, nucleotide changes in promoter regions, or inserted or deleted base (inser or del with position in coding sequence) resulting in a frameshift. Bold text shows mutations concordantly detected by whole-genome sequencing and Deeplex-MycTB (GenoScreen, https://www.genoscreen.fr) in samples subjected to both assays. Other mutations are those detected in samples analyzed by Deeplex-MycTB only. Drug resistance predictions are based on reference data from available scientific literature (*8–10*), and for *pncA* also on data from Yadon et al. (*14*). Black represents phenotypic resistance to the different drugs and gray represents phenotypic susceptibility. For phenotypic testing, levofloxacin was the only fluoroquinolone tested. AMI, amikacin; CAP, capreomycin; EMB, ethambutol; ETH, ethionamide; FQ, fluoroquinolones; KAN, kanamycin; INH, isoniazid; ND, not done; PZA, pyrazinamide; RIF, rifampin; SM, streptomycin. †Deeplex-MycTB result obtained on a primary specimen (sputum). The other results were obtained on indirect samples (primary cultures). ‡Mutation described in association with isoniazid resistance once before by Wang et al. (*11*). This mutation is not detectable by Anyplex testing. §Detected as a minority variant, at 5.2% in this sample (see text). Percentages of fixation of other mutations within individual samples range from 80.6% to 100%. ¶Putative deletion, as inferred by absence of reads mapped specifically on the corresponding gene target, in contrast to all other, well covered targets. #Internal deletion, resulting in a frameshift, from gene position 859 to 999. **According to MIRU-VNTRPlus website (http://www.miru-vntrplus.org) nomenclature (*15*). For patient 185, a question mark in the genotype reflects the absence of a detectable allele in locus 4052. ††According to Deeplex-MycTB (spoligotyping and phylogenetic SNPs) and MIRU-VNTRPlus identification, confirmed by whole-genome sequencing results, when done.

Results of genotypic analysis of the 3 MDR TB isolates by Deeplex-MycTB also were consistent with phenotypic profiling overall, considering that a rare F129S mutation in *katG* was previously described in association with isoniazid resistance (*11*), along with other well-established mutations. An ethambutol resistance–associated M306V mutation in *embB* in 1 isolate was phenotypically undetected, probably reflecting known poor phenotypic reproducibility for this mutation (*10*). Of note, in the same isolate, Deeplex-MycTB testing detected a *gyrA* S91P mutation, which generally confers low levels of levofloxacin resistance (*12*), as a minority population (5.2%). This detection was confirmed by Anyplex results but was not correlated with phenotypic resistance to levofloxacin tested at a standard critical concentration of 1.5 µg/mL. As with the XDR TB isolates, nonsense insertion or deletion mutations additionally detected in *pncA* or *ethA* predicted supplementary pyrazinamide and ethionamide resistance in some isolates.

None of the MDR or XDR TB cases clustered with any other case in the study population tested by standard 24-locus mycobacterial interspersed repetitive unit–variable-number tandem-repeat (MIRU-VNTR) typing of isolates, showing no support for drug-resistance transmission (Appendix 2). Consistently, 4 of the 6 cases involved were previously treated, and the 2 new cases were in migrant workers, presumably representing imported cases. Two cases were in Syria refugees; 1 patient with MDR TB had repeated failed treatment in Syria, and 1 XDR TB case was a relapse after patient arrival in Lebanon. Of the other previously treated cases, 2 had Beijing strain genotypes; the isolate from an XDR TB case in a patient originating from eastern Europe differed by a single allele from the 100-32 MIRU-VNTR haplotype and the isolate from an MDR TB case in a patient from Lebanon fully matched the 100-32 MIRU-VNTR haplotype (Table 2). This haplotype represents a major, presumably highly transmissible MDR-associated clonal complex epidemically spreading across Eurasia (*13*). Although an XDR TB patient of foreign origin returned to his country after diagnosis because of initial unavailability of proper treatment in Lebanon, the 2 other XDR TB patients received treatment and, as of January 2019, responded positively to ongoing treatments, as were the patients treated for MDR TB.

## Conclusions

Although the prevalence of rifampin-resistant TB estimated in Lebanon is relatively low (2.8%), identification of XDR TB and MDR TB cases, including TB strains with strong epidemic potential and complex resistance patterns, calls for sustained diagnosis of MDR TB. We recommend that Lebanon test all TB-positive isolates for resistance to first- and second-line drugs, to inform treatment decisions and prevent the spread of drug resistance. Other major destinations for refugees, asylum seekers, and migrant workers should also consider using enhanced diagnostics to help prevent the spread of drug-resistant TB.

Appendix 1 Additional methods for study of drug-resistant tuberculosis, Lebanon, 2016–2017.

Appendix 2 Additional information on individual cases in study of drug-resistant tuberculosis, Lebanon, 2016–2017.
