# Supplementary material for: Drug-Resistant Tuberculosis, Lebanon, 2016 – 2017
Source: Emerg Infect Dis. 2019 Mar;25(3):564–8. doi: 10.3201/eid2503.181375 (PMC6390733; doi:10.3201/eid2503.181375)
Supplement: Appendix 1 — Additional methods for study of drug-resistant tuberculosis, Lebanon, 2016–2017. [file 18-1375-Techapp-s1.pdf]

Article DOI: https://doi.org/10.3201/eid2503.181375

Drug-Resistant Tuberculosis, Lebanon, 2016–2017

Appendix 1

Methods

Patient Data Collection

A standardized form was used to collect patient data, comprising patient age, sex, nationality, TB history, in- or outpatient status, and year of birth. Data were anonymized for subsequent analysis.

Study Design and Population

Clinical samples for all suspected TB cases collected as part of the national TB program from June 1, 2016 through May 31, 2017, were included. Clinical samples from centers located in different governorates in Lebanon, except those in the Northern region, were centralized in the TB reference center in Karentina-Beirut. Then, samples were transferred twice a week, with help from the International Organization for Migration, for subsequent testing to the Laboratoire Microbiologie Santé et Environnement (LMSE) at the Azm Center for Research in Biotechnology and Its Applications at the Lebanese University in Tripoli. Samples from the local TB centers in the Northern region were sent directly to LMSE.

Routine Diagnostics

Confirmation of TB cases was performed in Lebanon as follows. First, microscopic examination was performed in the local TB centers by using Ziehl-Neelsen coloration with a fast cold staining, Kit Quick-TB (RAL diagnostics, http://www.ral-diagnostics.fr), or Cold Kinyoun Stain Kit (Atom Scientific, https://atomscientific.com). Xpert MTB/RIF (Cepheid, http://www.cepheid.com) tests were performed at the Karentina TB center in Beirut or at LMSE in Tripoli.

All samples transferred to LMSE were subjected to microscopic examination or reexamination, and then decontaminated by using standard NaOH/N-acetyl-cysteine treatment.

Primary culture was performed for all microscopy-positive and -negative samples at LMSE by using solid BBL Lowenstein-Jensen LJ (Beckton-Dickinson, https://www.bd.com) and liquid BBL Mycobacteria Growth Indicator Tube (MGIT) (Beckton-Dickinson, https://www.bd.com) media following manufacturer's instructions.

For samples contaminated with blood, samples from paraffin-embedded biopsies, or in case of Xpert-negative and culture-positive results, Anyplex MTB/NTM Real-time detection Kit (Seegene, http://www.seegene.com) was used to confirm identification of *M. tuberculosis* complex.

Phenotypic drug susceptibility testing was performed by using the BD MGIT SIRE (Becton Dickson, https://www.bd.com) liquid culturing kit at critical concentrations for isoniazid (0.1 mg/L), rifampin (1.0 mg/L), streptomycin (1.0 mg/L), or ethambutol (5 mg/L) and the BACTEC MicroMGIT Reader (Becton Dickson, https://www.bd.com) by following the manufacturer's recommendations. In cases of resistance to rifampin and isoniazid, testing was performed by using the same liquid culture system with critical concentrations for amikacin (1.0 mg/L), kanamycin (2.5 mg/L), and levofloxacin (1.5 mg/L) (Sigma Aldrich, https://www.sigmaaldrich.com).

In urgent cases, such as treatment relapse or rifampin resistance detection by Xpert MTB/RIF, Anyplex II MTB/MDR and Anyplex II MTB/XDR detection Kits (Seegene, http://www.seegene.com) were used for detection of mutations associated with isoniazid, rifampin, fluoroquinolones, and injectable drugs.

Whole-genome Sequencing

Whole-genome sequencing was performed at the WHO Collaborating Supranational TB Reference Center at San Raffaele Scientific Institute in Milan, Italy. Genomic DNA was extracted from cultured isolates by using the cetyl-trimethylammonium bromide (CTAB) method (*1*), and quantified by using the Qubit dsDNA BR assay (Life Technologies, ThermoFisher Scientific, https://www.thermofisher.com). Paired-end libraries of 100 bp read length were prepared using the Nextera XT DNA Sample Preparation kit (Illumina Inc., https://www.illumina.com) and sequenced on an Illumina HiSeq 2500 platform according to the manufacturer's instructions. DNA sequence files were deposited in the BioProject database (https://www.ncbi.nlm.nih.gov/bioproject) under accession code PRJNA488372. Downstream

analysis was performed by using a dedicated in-house bioinformatics pipeline in Milan, including quality control check, alignment to H37Rv reference genome, recalibration, and variant calling, as described in Tagliani, et al. (*2*). A mean read coverage depth of >30x, with at least 4 reads on forward and reverse strand, at least 4 allele calls with base quality ≥20, and allele frequency ≥50% were considered acceptable to call variants. The association of mutations with drug resistance was based on available scientific literature (*3*–*8*).

Targeted deep sequencing

A targeted sequencing approach was used as an alternative molecular test in Lille, France, by using a beta version of Deeplex-MycTB kit (GenoScreen, https://www.genoscreen.fr). Targeted sequencing is more tolerant of small amounts and lower integrity of mycobacterial DNA, which we found in samples transported under suboptimal conditions from Lebanon. Briefly, this assay uses deep sequencing of a single 24-plexed amplicon mix for simultaneous mycobacterial species identification (*hsp*65), genotyping (spoligotyping and phylogenetic SNPs) and prediction of drug resistance of *M. tuberculosis* complex strains. A total of 18 gene regions associated with resistance to first- and second-line drugs are included (Figure). DNA was extracted from heat inactivated clinical specimens or cultured isolates by using MasterPure DNA Purification Kit (Epicenter, Illumina, http://www.epibio.com/). Amplicons were purified by using AMPure XP (Agencourt, Beckman Coulter, https://www.beckmancoulter.com) magnetic beads and quantified by Qubit dsDNA BR assay (Life Technologies, https://www.thermofisher.com). Paired-end libraries of 150 bp read length next-generation sequencing were prepared as described above and sequenced on an Illumina MiSeq platform according to the manufacturer’s instructions. DNA sequence files were deposited in the BioProjet database under accession code PRJNA488592. Variant calling and genotypic analysis was performed by using a dedicated, parameterized software developed by GenoScreen (https://www.genoscreen.fr).

MIRU-VNTR typing

Standard 24-locus MIRU-VNTR typing was performed as described in Supply et al. (*9*) by using MIRU-VNTR Typing kits for amplification with 6 quadruplex PCRs (Genoscreen, https://www.genoscreen.fr). The sizes of the amplified fragments and the numbers of repeats in the target loci were determined after capillary electrophoresis-based separation on an ABI 3730 XL DNA Analyzer (ThermoFisher Scientific, Applied BioSystems, www.thermofisher.com),

using a customized software, GeneMapper v.5, (ThermoFisher Scientific, Applied BioSystems, https://www.thermofisher.com). To perform cluster analysis and prediction of genetic lineage of isolates, the genotypes were analyzed and compared with reference strain genotypes by using tools implemented in the MIRU-VNTRplus database (http://www.miru-vntrplus.org), as described in Weniger, et al. (*10*) and Allix-Béguec, et al. (*11*). Genotyping analysis was made blinded from microbiological data and patient data.

Statistical Analysis

Statistical analysis was conducted by using SAS v9.4 software (SAS Institute Inc., https://www.sas.com). Age was expressed as mean ±SD, categorical variables were expressed as absolute numbers and percentages. Multivariate logistic regression was used to test TB history as an independent predictor of drug resistance, after adjustment for age, sex, and nationality (Appendix 2). The log-linearity assumption was checked for the continuous covariate (patient age). The multivariate model was built by first including all predictors and then using a manual backward selection to reduce the model, minimizing Schwarz's Bayesian Criterion and maximizing the c-statistics and the p-value of Hosmer-Lemeshow test. A two-tailed type I error rate of 5% was considered for statistical significance.

References

1. van Soolingen D, Hermans PW, de Haas PE, Soll DR, van Embden JD. Occurrence and stability of insertion sequences in *Mycobacterium tuberculosis* complex strains: evaluation of an insertion sequence-dependent DNA polymorphism as a tool in the epidemiology of tuberculosis. J Clin Microbiol. 1991;29:2578–86. PubMed

2. Tagliani E, Hassan MO, Waberi Y, De Filippo MR, Falzon D, Dean A, et al. Culture and next-generation sequencing-based drug susceptibility testing unveil high levels of drug-resistant-TB in Djibouti: results from the first national survey. Sci Rep. 2017;7:17672. PubMed http://dx.doi.org/10.1038/s41598-017-17705-3

3. Whitfield MG, Soeters HM, Warren RM, York T, Sampson SL, Streicher EM, et al. A global perspective on pyrazinamide resistance: systematic review and meta-analysis. PLoS One. 2015;10:e0133869. PubMed http://dx.doi.org/10.1371/journal.pone.0133869

4. Miotto P, Cabibbe AM, Feuerriegel S, Casali N, Drobniewski F, Rodionova Y, et al. *Mycobacterium tuberculosis* pyrazinamide resistance determinants: a multicenter study. MBio. 2014;5:e01819–14. PubMed http://dx.doi.org/10.1128/mBio.01819-14

5. Ramirez-Busby SM, Valafar F. Systematic review of mutations in pyrazinamidase associated with pyrazinamide resistance in *Mycobacterium tuberculosis* clinical isolates. Antimicrob Agents Chemother. 2015;59:5267–77. PubMed http://dx.doi.org/10.1128/AAC.00204-15

6. Feuerriegel S, Schleusener V, Beckert P, Kohl TA, Miotto P, Cirillo DM, et al. PhyResSE: a web tool delineating *Mycobacterium tuberculosis* antibiotic resistance and lineage from whole-genome sequencing data. J Clin Microbiol. 2015;53:1908–14. PubMed http://dx.doi.org/10.1128/JCM.00025-15

7. Farhat MR, Sultana R, Iartchouk O, Bozeman S, Galagan J, Sisk P, et al. Genetic determinants of drug resistance in *Mycobacterium tuberculosis* and their diagnostic value. Am J Respir Crit Care Med. 2016;194:621–30. PubMed http://dx.doi.org/10.1164/rccm.201510-2091OC

8. Miotto P, Tessema B, Tagliani E, Chindelevitch L, Starks AM, Emerson C, et al. A standardised method for interpreting the association between mutations and phenotypic drug resistance in *Mycobacterium tuberculosis.* Eur Respir J. 2017;50:1701354. PubMed http://dx.doi.org/10.1183/13993003.01354-2017

9. Supply P, Allix C, Lesjean S, Cardoso-Oelemann M, Rüsch-Gerdes S, Willery E, et al. Proposal for standardization of optimized mycobacterial interspersed repetitive unit-variable-number tandem repeat typing of *Mycobacterium tuberculosis*. J Clin Microbiol. 2006;44:4498–510. PubMed http://dx.doi.org/10.1128/JCM.01392-06

10. Weniger T, Krawczyk J, Supply P, Niemann S, Harmsen D. MIRU-VNTRplus: a web tool for polyphasic genotyping of *Mycobacterium tuberculosis* complex bacteria. Nucleic Acids Res. 2010;38(Suppl):W326-W331. PubMed http://dx.doi.org/10.1093/nar/gkq351

11. Allix-Béguec C, Harmsen D, Weniger T, Supply P, Niemann S. Evaluation and strategy for use of MIRU-VNTRplus, a multifunctional database for online analysis of genotyping data and phylogenetic identification of *Mycobacterium tuberculosis* complex isolates. J Clin Microbiol. 2008;46:2692–9. PubMed http://dx.doi.org/10.1128/JCM.00540-08
